# Scoring Tool to Predict Need for Early Video-Assisted Thoracoscopic Surgery (VATS) After Pediatric Trauma

**DOI:** 10.1007/s00268-023-07141-y

**Published:** 2023-08-31

**Authors:** Brian Kazempoor, Jeffry Nahmias, Isabel Clark, Sebastian Schubl, Michael Lekawa, Lourdes Swentek, Hari B. Keshava, Areg Grigorian

**Affiliations:** 1 Department of Surgery, University of California, Irvine, Orange, CA USA; 2 Division of Trauma, Burns and Surgical Critical Care, Department of Surgery, University of California, Irvine Medical Center, 333 The City Blvd West, Suite 1600, Orange, CA 92868-3298 USA

## Abstract

**Background:**

No widely used stratification tool exists to predict which pediatric trauma patients may require a video-assisted thoracoscopic surgery (VATS). We sought to develop a novel VATS-In-Pediatrics (VIP) score to predict the need for early VATS (within 72 h of admission) for pediatric trauma patients.

**Methods:**

The pediatric 2017–2020 Trauma Quality Improvement Program database was used and divided into two sets (derivation set using 2017–2019 data and validation set using 2020 data). First, multiple logistic regression models were created to determine the risk of early VATS for patients ≤ 17 years old. Second, the weighted average and relative impact of each independent predictor were used to derive a VIP score. We then validated the score using the area under the receiver operating characteristic (AROC) curve.

**Results:**

From 218,628 patients in the derivation set, 2183 (1.0%) underwent early VATS. A total of 8 independent predictors of VATS were identified, and the VIP score was derived with scores ranging from 0 to 9. The AROC for this was 0.91. The VATS rate increased steadily from 12.5 to 32% then 60.5% at scores of 3, 4, and 6, respectively. In the validation set, from 70,316 patients, 887 (1.3%) underwent VATS, and the AROC was 0.91.

**Conclusions:**

VIP is a novel and validated scoring tool to predict the need for early VATS in pediatric trauma. This tool can potentially help hospital systems prepare for pediatric patients at high risk for requiring VATS during their first 72 h of admission. Future prospective research is needed to evaluate VIP as a tool that can improve clinical outcomes.

## Introduction

Nearly 15% of children in the USA present annually to the hospital with a traumatic injury [1]. Most pediatric patients are found to have nonsignificant injuries and are discharged home within 24 h [2]. Of patients that have significant trauma, nearly 30% may have a thoracic injury with the most common being a lung injury [3].

Hemodynamically stable trauma patients with thoracic injuries requiring intervention are often managed with only tube thoracostomy. However, in 20% of cases, a thoracostomy will be insufficient and surgical intervention will be required [4]. This may be due to on-going bleeding, persistent pneumothorax, air-leak, empyema, or diaphragmatic injury [5]. The most common indication for thoracic surgical intervention in trauma patients with a chest tube is a retained hemothorax [5]. In adults, video-assisted thoracoscopic surgery (VATS) is used in over 30% of cases with retained hemothorax [6]. VATS has also been used safely in children for a variety of conditions including empyema and pneumothorax [7, 8].

Performing VATS earlier in the hospital course has been demonstrated to be associated with improved outcomes in adult trauma patients. The Eastern Association for the Surgery of Trauma (EAST) guidelines recommends early VATS within the first 72 h after injury for patients with retained traumatic hemothorax to decrease the need for additional procedures, conversion to open thoracotomy, empyema, and to reduce hospital length of stay (LOS) [9]. A retrospective study of over 80 patients found that VATS performed ≤ 5 days post-injury in adult patients with blunt chest trauma was associated with a significantly lower thoracotomy conversion rate and shorter hospital LOS [10]. Another study on adults with thoracic trauma found that those receiving VATS ≥ 5 days post-injury had higher rates of surgical site infection, unplanned intubation, and pneumonia [11]. Thus, there is substantial utility in predicting the need for VATS in all trauma patients, as performing early VATS appears to be associated with a decreased rate of complications and LOS.

Given the potential benefits of early VATS in trauma patients, there is a pressing need to develop a predictive tool for determining which pediatric trauma patients may require early VATS intervention. Currently, no widely used stratification tool exists for this purpose. To address this gap in the literature and improve pediatric trauma outcomes and hospital system readiness, we sought to develop and validate a novel VATS-In-Pediatrics (VIP) score. The objective of our study is to predict the need for early VATS within 72 h of admission for pediatric trauma patients using the VIP score.

## Material and methods

This study was deemed exempt from our local Institutional Review Board as it utilized a national de-identified database. The 2017–2019 Trauma Quality Improvement Program (TQIP) database was queried to develop the VIP score. The TQIP database is a national database created by the American College of Surgeons to compile and collect comprehensive prospective trauma data [12]. All pediatric trauma patients (≤ 17 years old) presenting to the emergency department were included for analysis. The primary outcome was undergoing early VATS within 72 h of arrival. Exclusion criteria included patients with conversion of thoracoscopic to open surgery. After developing the VIP scoring tool, we used the 2020 TQIP database to validate the VIP scoring tool. The dataset was separated into two groups: those that underwent VATS within 72 h of arrival versus those that did not undergo VATS.

The VIP score was then derived using a three-step methodology, which has been validated in the literature [13–16]. First, univariate analyses of known risk factors for early VATS were performed. These variables were selected following a detailed literature review and consensus among authors [10, 11]. We selected variables that are readily accessible after initial trauma workup is completed and that are consistently reported within the TQIP database to allow for generalizability and expedited use of the scoring tool. The variables examined included age, injury severity score (ISS), vitals on admission (hypotension, tachypnea, tachycardia), length of stay (LOS), mechanism of injury, and types of injury. A univariate analysis was used to identify potential risk factors for early VATS. Variables with a *p* value < 0.2 were then included in a stepwise multivariable forward logistic regression model to identify independent risk factors for early VATS. The odds ratio of each risk factor was then used to assess the relative impact and assign a point value. This point value was derived by dividing by the lowest common denominator (i.e., the lowest odds ratio) and rounding to the nearest whole integer. Numerous iterations were developed to account for small differences in rounding and error. For each iteration, a receiver operating curve was created. The area under the receiver operating characteristic (AROC) curve was then examined to assure consistency. In this study, the AROC measures the ability of the model to discriminate between the occurrence of early VATS versus its absence. Finally, the validation set was used to test the VIP score generated from the derivation set. After deriving the VIP scoring tool, validation was performed using the 2020 TQIP dataset, using the same criteria. All analyses were performed with IBM SPSS Statistics for Windows (Version 28, IBM Corp., Armonk, NY).

## Results

From 218,628 pediatric trauma patients, a total of 2183 patients (1.0%) underwent early VATS. In the derivation set, the early VATS group was older (median age, 16 vs. 10, *p* < 0.001), compared to the non-VATS cohort. The most common mechanisms of injury for patients undergoing early VATS were gunshot wound (39.0%) and motor vehicle collision (34.6%) [Table 1]. The most prevalent injuries for patients undergoing early VATS were to the lung (41.0%), small intestine (33.9%), and liver (31.4%) [Table 2]. From the patients in the derivation set, 1.1% of patients underwent VATS within 24 h, 0.1% underwent VATS within 24–72 h, and 0.1% underwent VATS after 72 h.

**Table 1:** Demographics of pediatric trauma patients

Characteristic	No VATS^a^	Early VATS^a^	*p* value
	(*n* = 216,445)	(*n* = 2183)	
Age, years, median (IQR)	10 (5, 15)	16 (12, 17)	<0.001
ISS^b^, median (IQR)	4 (2, 9)	17 (9, 30)	<0.001
Male, *n* (%)	139,176 (64.2%)	1402 (74.3%)	<0.001
*Vitals on Admission, n (%)*			
Hypotension (SBP^c^ < 90 mmHg)	5178 (2.7%)	369 (17.3%)	<0.001
Tachypnea (≥ 22/RPM^d^)	89,922 (43.1%)	929 (44.9%)	0.710
Tachycardia (≥ 120/BPM^e^)	46,574 (22.1%)	689 (32.1%)	<0.001
*Comorbidities, n (%)*			
ADHD^f^	8892 (4.1%)	100 (4.6%)	0.240
Hypertension	422 (0.2%)	7 (0.3%)	0.181
Mental/Personality disorders	4642 (2.2%)	81 (3.8%)	<0.001
Current smoker	3350 (1.6%)	129 (6.0%)	<0.001
Substance abuse disorders	1988 (0.9%)	89 (4.1%)	<0.001
*Blunt Mechanism, n (%)*			
Bicycle	10,584 (4.9%)	68 (3.1%)	<0.001
Fall	81,561 (37.7%)	61 (2.8%)	<0.001
Motorcycle	4050 (1.9%)	28 (1.3%)	0.043
Motor vehicle collision	49,799 (23.0%)	756 (34.6%)	<0.001
Auto vs. pedestrian	11,266 (5.2%)	105 (4.8%)	0.408
*Penetrating Mechanism, n (%)*			
Gunshot wound	8757 (4.0%)	852 (39.0%)	<0.001
Stab wound	6277 (2.9%)	231 (10.6%)	<0.001

^a^Video-assisted thoracoscopic surgery

^b^Injury severity score

^c^Systolic blood pressure

^d^Respirations per minute

^e^Beats per minute

^f^Attention deficit hyperactivity disorder

**Table 2:** Injuries in pediatric trauma patients

Injury, *n* (%)	No VATS^a^	Early VATS^a^	*p* value
	(*n* = 216,445)	(*n* = 2183)	
Rib fracture	6356 (2.9%)	413 (18.9%)	<0.001
Cardiac	335 (0.2%)	80 (3.7%)	<0.001
Diaphragm	248 (0.1%)	279 (12.8%)	<0.001
*Gastrointestinal tract*			
Esophagus	87 (<0.1%)	6 (0.3%)	<0.001
Stomach	166 (0.1%)	211 (9.7%)	<0.001
Small intestine	966 (0.4%)	740 (33.9%)	<0.001
Colon	686 (0.3%)	602 (27.6%)	<0.001
Rectum	189 (0.1%)	169 (3.2%)	<0.001
Lung	15,500 (7.2%)	896 (41.0%)	<0.001
*Organ*			
Bladder	270 (0.1%)	85 (3.9%)	<0.001
Kidney	2464 (1.1%)	334 (15.3%)	<0.001
Liver	4851 (2.2%)	686 (31.4%)	<0.001
Spleen	4409 (2.0%)	464 (21.3%)	<0.001

^a^Video-assisted thoracoscopic surgery

**Fig. 1:**
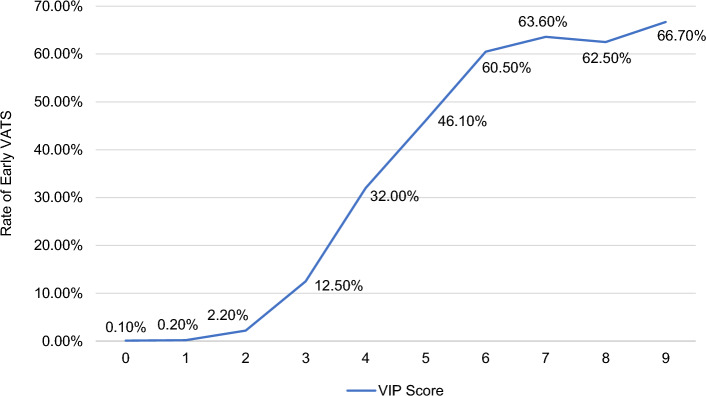
Rate of early video-assisted thoracoscopic surgery (VATS) in the pediatric trauma population for various VATS-In-Pediatrics (VIP) scores

**Fig. 2:**
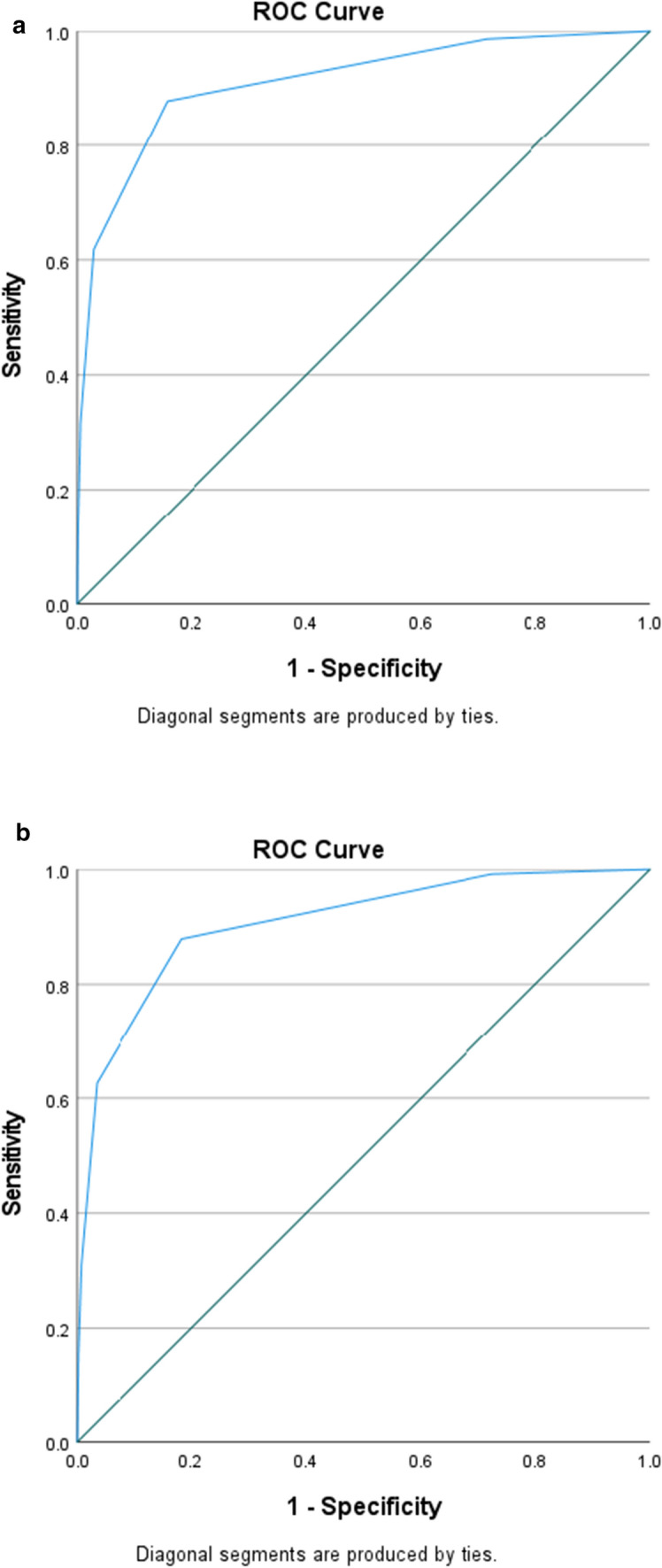
Area under the curve for development of the VATS-In-Pediatrics scoring tool **a** Test set [AROC = 0.91] **b** Validation set [AROC = 0.91]

Multiple stepwise logistic regression identified a total of 8 independent predictors for early VATS, and the VIP score was derived with scores ranging from 0 to 9. The predictors consisted of demographic (male sex and hypotension on admission), mechanism (penetrating), and injury variables (diaphragm, lung, cardiac, gastrointestinal tract, organ). As a group, injury was the predominant predictor (accounts for 67% of the total score), followed by demographics (22%) and penetrating mechanism (11%). The strongest individual independent predictor was cardiac injury (2 points) [Table 3]. The VATS rate increased steadily from 12.5 to 32% then 60.5% at scores of 3, 4, and 6, respectively [Fig. 1]. The AROC for the development of the VIP scoring tool was 0.91 [Fig. 2a]. In the validation set, from 70,316 patients, 887 (1.3%) underwent VATS, and the AROC was 0.91 [Fig. 2b].

**Table 3:** Development of the VATS-In-Pediatrics scoring tool

Variable	Points
*Demographics*	
Male	1
Hypotension on admission (SBP^a^ < 90 mmHg)	1
*Mechanism*	
Penetrating	1
*Injury*	
Diaphragm	1
Lung	1
Organ (bladder, kidney, liver, spleen)	1
Cardiac	2
Gastrointestinal tract	1
Maximum score	9
ROC^b^	0.91
95% CI for ROC^b^	0.90–0.92

^a^Systolic blood pressure

^b^Receiver operating characteristic

We then identified 5,147 patients with AIS grade ≥ 3 for the thorax. We found the AROC for the VIP scoring tool was 0.847 and 5.3% of this subset of patients underwent VATS within 24 h. We then performed another subset analysis in patients with an AIS grade ≥ 2 for the thorax. We found the AROC for this was 0.851 and 4.0% of these patients underwent VATS within 24 h.

## Discussion

In this national analysis, we found that 1% of pediatric trauma patients underwent early VATS. Older pediatric patients were more likely to undergo early VATS likely due to the incidence of penetrating mechanism injuries and motor vehicle collisions resulting in polytrauma increasing considerably with older age [17, 18]. We identified a novel and clinically relevant VIP scoring tool to predict the need for early VATS within 72 h of hospital arrival. This tool can potentially be used to allocate appropriate resources and mitigate complications related to delays in timely surgical intervention, and appears to be more appropriate for all trauma patients rather than those specifically with thoracic injuries.

The VIP score is comprised of 8 variables with the majority being injuries. A cardiac injury was the strongest predictor for early VATS. In the case of penetrating trauma, a cardiac injury and adjacent pericardiotomy can lead to bleeding into the thoracic cavity. If the bleeding subsides and the chest tube does not evacuate all thoracic contents, a retained hemothorax may necessitate VATS. In a study of nearly 300 pediatric patients with a traumatic hemothorax, nearly 80% of patients were successfully managed with VATS [19]. Similarly in adults, a meta-analysis of over 450 patients demonstrated that early VATS was associated with a higher success rate and shorter hospital LOS [20]. However, we must emphasize the potential of cardiac injury as a marker for severe thoracic trauma, and so the concurrent nature of multiple thoracic injuries may be the indication for VATS instead of the cardiac injury in and of itself. Lung and diaphragm injuries are other important components to the VIP score. Nearly half of the patients undergoing early VATS had a lung injury and more than 10% had a diaphragm injury. Both these injuries can be managed with a minimally invasive approach including VATS. Injuries to the gastrointestinal tract and organs also comprised the VIP score. It is likely that most of these patients did not present with isolated abdominal trauma but rather with concurrent thoracic trauma. However, certain abdominal injuries complicated by perforated viscous coupled with a diaphragm rupture/laceration can cause subsequent complications in the thorax (i.e., empyema), which may necessitate early VATS [21]. And finally, male pediatric patients’ propensity for early VATS is likely explained by their engagement in higher-risk behaviors as compared to females [22]. This may have led to more severe injuries requiring intervention, and likely explains why we found sex to be a factor in the VIP scoring tool.

When required, performing VATS earlier during the hospitalization is associated with a decreased risk of complications and shorter LOS. This has been demonstrated in both adult and pediatric populations [11, 23]. The VIP scoring tool may potentially help trauma teams accurately predict which trauma patients will require early VATS based on vitals, mechanism, and injuries, all of which are readily available within the first few hours of arrival. This can help hospital and trauma systems allocate resources in a timely manner and reduce the rate of complications associated with delayed intervention [24–26]. Scoring tools have been used to utilize limited hospital resources more efficiently [27, 28]. The VIP score can potentially assist hospital systems in preparing for pediatric patients that may require prolonged inpatient care and additional, unforeseen interventions or treatments. However, the utility of this tool will need to be validated with future prospective research.

There are several limitations to our study. As a large, retrospective national database study, our data are subject to reporting bias, misclassification, and missing data. We were not able to assess indications for VATS or evaluate granular operative data to analyze the precise procedures performed, intraoperative findings, or hemodynamic stability of the patient during the operation. Although our study did not specifically analyze the potential influence of physician specialty or experience, we acknowledge it is plausible that adult trauma surgeons might have a different threshold for utilizing VATS compared to pediatric surgeons. For instance, adult trauma surgeons may be more accustomed to managing more severe injuries and may therefore be more likely to opt for surgical interventions like VATS. Conversely, pediatric surgeons could potentially favor conservative management. However, it is also crucial to note that the decision to perform VATS or to opt for a more conservative approach is multifactorial and not solely dependent on the surgeon’s specialty. Factors such as the patient’s overall condition, the nature and severity of the injury, and the presence of other injuries would likely play a significant role in this decision. Additionally, the VIP score was developed using a specific set of variables, and it is possible that other factors not included in the model may also contribute to the need for early VATS. However, given the high discriminative ability of the VIP score (AROC = 0.91), it is likely that the included variables capture the majority of the relevant factors for predicting early VATS requirement. Our scoring tool requires further prospective validation and assessment for its clinical utility.

Clinical algorithms can provide a framework, but they are not substitutes for comprehensive patient assessment and individualized care. We developed the scoring system to assist in identifying pediatric patients at high risk for requiring VATS after trauma, based on readily available data points. It is not intended to dictate clinical care, but rather to provide some guidance in a complex clinical scenario. We acknowledge that there are many nuances of patient care and other clinical features and circumstances that the VIP score does not capture. The score is meant to be a supplementary tool for clinicians, providing additional information that can be used in conjunction with their own expertise and judgment. Additionally, the VIP score can be used as a quality control measure to ensure optimal outcomes for patients treated at a trauma center.

## Conclusions

VIP is a novel and validated scoring tool derived from a large national database that predicts the need for early VATS in pediatric trauma during the first 72 h of admission. This tool can potentially help improve hospital system readiness and better allocate availability of resources. However, the VIP scoring tool warrants further prospective validation and assessment for its clinical utility to improve pediatric trauma outcomes.
